# Discovery of EEG effective connectivity during visual motor imagery with multi-scale symbolic transfer entropy

**DOI:** 10.1038/s41598-025-22143-7

**Published:** 2025-10-31

**Authors:** Haobo Zhou, Keiji Iramina

**Affiliations:** 1 Graduate School and Faculty of Information Science and Electrical Engineering, Kyushu University, Fukuoka, 819-0382 Japan; 2 Faculty of Information Science and Electrical Engineering, Kyushu University, Fukuoka, 819-0382 Japan

**Keywords:** Motor Imagery, EEG, Connectivity Network, Transfer Entropy, Neuroscience, Psychology

## Abstract

Visual motor imagery (VMI) is an important component of motor imagery, with potential applications in brain-computer interfaces and motor rehabilitation due to its lower training cost compared to kinesthetic motor imagery (KMI). However, the neural mechanisms underlying VMI, particularly the effects of imagery hand and imagery perspective (first-person perspective, 1pp, vs. third-person perspective, 3pp) remain unclear. This study examines the effective connectivity of VMI EEG using multi-scale symbolic transfer entropy. Time-frequency analysis revealed prominent event-related synchronization (ERS) in the alpha and high-beta bands, while connectivity analysis emphasized strong information flow within the parieto-occipital network. Notably, hand effect dominant information flows were found between the motor and posterior parietal-occipital regions, while perspective suggested a more remarkable effect. 1pp imagery significantly enhanced top-down modulation of the occipital cortex, whereas 3pp imagery engaged the right posterior parietal region, suggesting stronger spatial localization processing. These findings provide novel insights into the distinct neural mechanisms of VMI and its potential applications in cognitive neuroscience and brain-machine engineering.

## Introduction

Motor imagery (MI) has become a prominent paradigm in the brain-computer interface (BCI) field due to its natural logic of use and potential for extensive applications. MI involves the mental simulation of an action without producing any actual motor output^1^. It can generally be divided into two types: kinesthetic motor imagery (KMI) and visual motor imagery (VMI). KMI requires individuals to imagine the sensation of muscle contractions during a specific action, whereas VMI requires them to visualize the action as if observing a corresponding image in their mind^2^. Furthermore, VMI can be subdivided based on the perspective of the imagined action: first-person-perspective VMI (VMI-1), in which participants envision the action from their own viewpoint, and third-person-perspective VMI (VMI-3), in which they observe their actions from an external point, like they are watching someone else’s behavior^3^.

Over the past decades, neuroimaging studies have highlighted distinctions in brain activation among different MI modalities, particularly regarding KMI. KMI has been shown to primarily engage regions such as the supplementary motor area (SMA), the dorsal and ventral premotor cortex (PMC), the primary motor cortex (M1), the superior and inferior parietal lobules, and sensorimotor areas. In contrast, VMI has been reported to involve regions less directly related to movement, including the precentral gyrus, posterior parietal lobe, and occipital areas^2–5^, as well as different subregions of the parietal lobe depending on the perspective^6–8^. Nevertheless, inconsistencies across experimental protocols and interpretations have made KMI the default focus for studying MI-related brain activity and developing subsequent applications, such as MI-based BCI systems. A commonly held view is that KMI resembles actual motor execution, centered on motor cortex-related areas of the frontal-parietal network, whereas VMI shares more similarities with motor observation, evidenced by stronger involvement of posterior parietal-occipital regions^9–11^.

Because of its ability to capture signal with high temporal resolution, noninvasive electroencephalography (EEG) enables the detailed investigation of MI-related time-frequency characteristics. The widely reported premovement event-related desynchronization (ERD) and postmovement event-related synchronization (ERS) in the contralateral motor cortex during KMI are considered among the most distinctive features of MI. These features have also been extensively applied in motor rehabilitation and human-machine interface research. However, whether VMI exhibits similar EEG characteristics is still under debate. In recent years, functional connectivity analyses have gained prominence for revealing topological relationships among brain regions, and directed connectivity methods–providing information on the direction of information flow–have been employed to explore interactions between brain regions during MI. For KMI, both unidirectional and bidirectional connectivity have been identified in frontal-parietal regions, including strong connections from the dorsal premotor cortex (dPMA) to M1, from SMA to M1, and from ventral premotor cortex (vPMA) to bilateral superior and inferior parietal lobules^12–15^. Nevertheless, the effective network dynamics of VMI remain unclear, making it challenging to characterize the precise nature of VMI and how it is represented in brain activity.

As research on MI expands in the BCI domain, deeper investigations into its physiological and psychological mechanisms have somewhat lagged. One key question is whether VMI, given its flexibility and lower cost, might replace KMI in practical contexts or whether it is better suited for applications that leverage its unique neural characteristics. Before addressing these questions, it is essential to elucidate the electrophysiological bases of VMI.

On the one hand, previous studies of motor imagery have predominantly focused on the motor cortex and related regions, often treating motor execution as a baseline for MI. Although this method is applicable to KMI, it may be less suitable for investigating VMI. Previous studies have suggested that motor cortex excitability is associated with KMI but not necessarily with VMI^16,17^, indicating that evaluating VMI through sensorimotor rhythms may yield limited or inconclusive results. Therefore, this study centers on parietal-occipital areas, frequently mentioned in earlier reports, to investigate how motor, spatial, and visual information is integrated during VMI guided by visual materials. On the other hand, recognizing the limitations of traditional methods such as dynamic causal modeling and Granger causality, we adopt a data-driven, nonlinear approach–multi-scale transfer entropy–to evaluate the causal EEG networks related to VMI. We also consider the main effects of the hand (left vs. right) and the perspective (VMI-1 vs. VMI-3). Finally, although our primary goal is to elucidate parietal-occipital network activity during VMI, we also investigate the typical time-frequency characteristics of VMI. These findings will help clarify the unique brain dynamics underlying VMI and differentiate them from those associated with KMI.

## Methods

### Subjects

A total of 17 subjects participated in this experiment (3 females, mean age: 24.3±1.9), all of whom were right-handed, had normal vision, and with no history of psychiatric illness. Handedness of subjects were evaluated by the Edinburgh Handedness Inventory (EHI). The average score was 89.7 and the standard deviation was 13.3. Before the experiment, practice sessions were conducted to ensure participants fully understood the task. After the experiment, participants completed the Vividness of Movement Imagery Questionnaire-2^18^ to assess their visual motor imagery (VMI) ability. Data from subjects who scored too low will be excluded from subsequent analyses. This study was conducted in accordance with the ethical principles of Kyushu university and the Declaration of Helsinki. All experimental protocols were approved by Kyushu university (ISEE 2024-12). Informed consent was obtained from all subjects or their legal guardian(s).

### Experimental paradigm

**Fig. 1:**
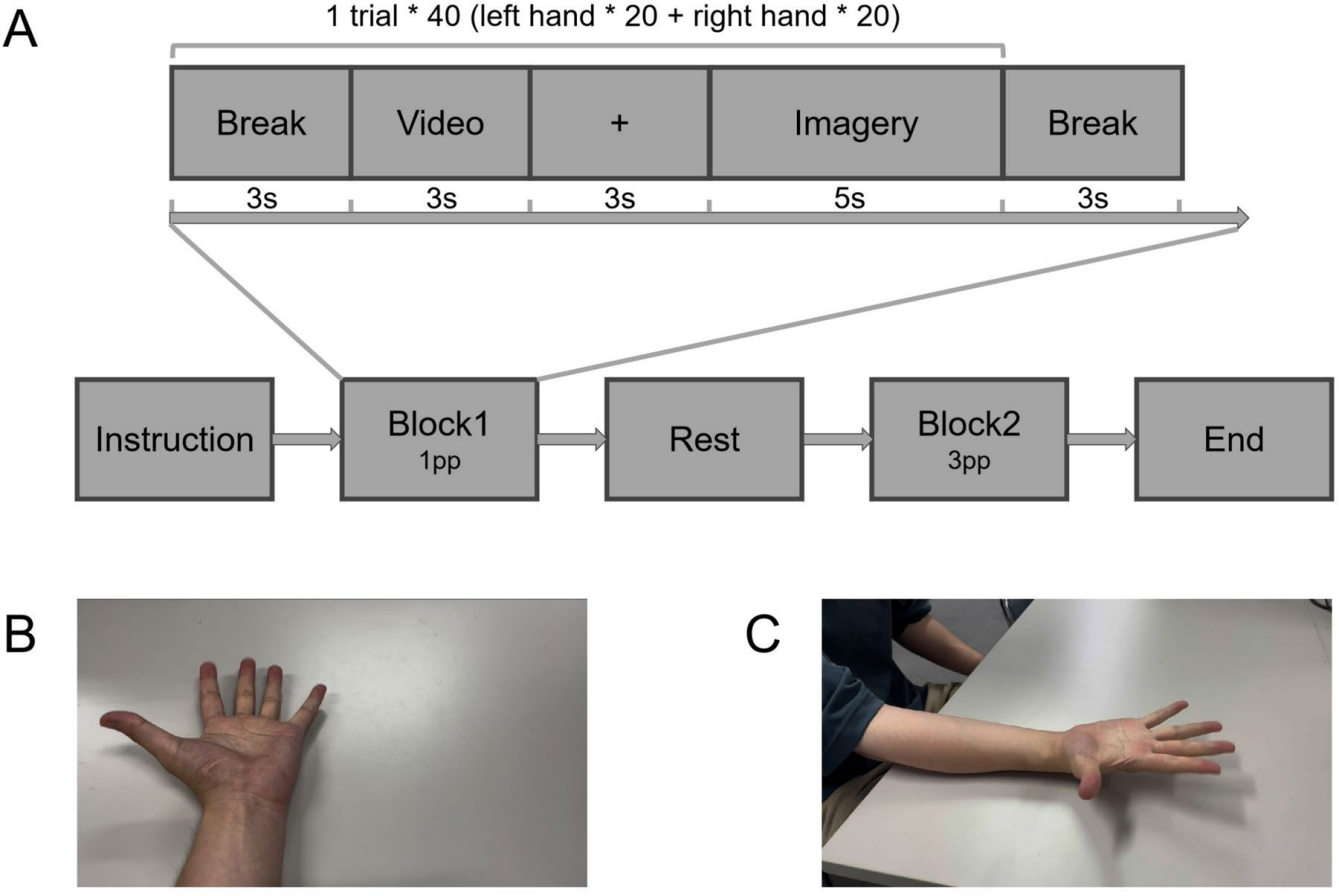
(**A**) The whole experiment procedure. (**B**) Cue material in 1pp block tasks (left hand). (**C**): Cue material in 3pp block tasks (right hand).

The experimental procedure consisted of two blocks. In the first block, subjects were required to complete a first-person perspective visual motor imagery task. Specifically, after a break between trials, a three-second video was presented on the screen, randomly showing the action of alternately opening the palm and making a fist with either the left or right hand from a first-person perspective (1pp). Then after a three-second fixation cross, subjects were required to perform imagery of the same movement as the condition just cued by the video during a five-second imagery period until a rest cue appeared on the screen. The second block of the experiment followed the same procedure as the first, except that the cue videos and imagery tasks presented the same hand movements from a third-person perspective (3pp). The imagery tasks were randomly repeated 20 trials for each condition in each block, for a total of 80 trials for the entire experiment (Fig.1). During the imagery period, subjects were asked to keep their eyes open, and their hands relaxed on their thighs. Between the two blocks, subjects had at least 3 minutes to rest and relax.

### EEG measurement and preprocessing

In this experiment, EEG data were recorded using a 64-channel system (Nihon Kohden EEG-2100) following the 10–10 electrode placement system, with a sampling rate of 1000 Hz. The reference electrode was placed on the right earlobe. Offline preprocessing was conducted for each participant’s data, including common average re-referencing, band-pass filtering (0.1–50 Hz), and artifacts removing. Specifically, independent component analysis (ICA) was applied using 15 components, a sampling frequency of 1000 Hz, and the FastICA^19^ algorithm to remove artifacts caused by eye movements, cardiac activity, and muscle activity. Artifact components were identified through manual inspection based on the time series, power spectral characteristics, and topographic maps of each component, using the ICA visualization tools provided in the MNE toolbox (version 1.6.1). Based on the distribution of the main brain regions activated during VMI reported in previous studies, including the vicinity of the motor cortex, posterior parietal regions, and occipital regions, C3, C4, P5, P6, O1, and O2 were selected as the electrodes of interest for the subsequent analyses in this experiment.

### Event-related desynchronization/synchronization

Power spectrum ERD/ERS are often used to reflect trends in the synchronization of cortical neuronal activity in the region of interest during a task, which is defined as the percentage increase/decrease in mean power during the event relative to the baseline period^20^:

$$RA(\%) = \left( \frac{\text {Act} - R}{R} \right) \times 100\%$$

where *Act* is the power value in event-related period, *R* is the average power in baseline period and *RA* is the relative amplitude. The baseline period in this study was set as 2s to 1s before the start of the imagery period.

### Multi-scale analysis

In the entropy analysis of EEG, the method of multi-scale analysis is often used to coarse-grain the raw time series. Different degrees of coarsening allow us to capture useful information at different time scales. The multi-scale approach based on sampling point averaging has been shown to be a scheme that can effectively represent the dynamic differences and is applicable to the analysis of biological time series^21^. It is defined as

$$y_j^{(\tau )} = \frac{1}{s} \sum _{i=(j-1)s+1}^{js} x_i, \quad 1 \le j \le \frac{N}{s}$$

where $y_{j}$ is the point in the coarse-grained series, *s* is the scale factor and $x_{i}$ is the element of the original time series. A smaller scale factor indicates that the coarse-grained signal is closer to the original signal, thus it retains more detailed information in the original signal. Whereas a larger scale factor indicates more neglect of detailed information and captures broader trends.

### Phase space reconstruction / permutation symbolization

Phase space reconstruction (PSR) is a commonly used method to characterize the nonlinear dynamics of a time series by reconstructing the equivalent attractor in phase space^22,23^. Given a discrete time series,

$$\{X(i) \mid i = 1,2, \dots , n\}$$

it is defined as:

$$X_k (i) = [x(i), x(i+\tau ), \dots , x(i+(m-1)\tau )]$$

where *m* is the embedding dimension, denoting the number of dimensions in the reconstructed phase space. In this study, *m* is chosen to be 3 to balance the complexity of the system and the limitation of the sequence length^24^. $\tau$ is the delay factor, which indicates the interval at which the reconstructed sequence coordinates are chosen to be interpolated. Too small a value of $\tau$ will result in the loss of information about the dynamics of the original system during the reconstruction process, while too large a value of $\tau$ will result in redundancy of information during the reconstruction. In this study, $\tau$ was determined by the autocorrelation function method^25^. *k* is the number of reconstructed components, equal to $n-(m-1)\tau$.

After obtaining a high-dimensional PSR sequence, it is usually encoded in combination with the permutation symbolization^26,27^ method to obtain a state sequence that can represent its dynamics information. For the *j*th component of the reconstructed sequence, firstly, arrange the elements in ascending order as:

$$x(i+(j_1-1)\tau ) \le x(i+(j_2-1)\tau ) \le \dots \le x(i+(j_m-1)\tau )$$

Then the order of subscript index value of each element is extracted as the state representation of the current component as:

$$S_j = \{ j_1, j_2, \dots , j_m \}$$

In this experiment, since the embedding dimension *m* is chosen to be 3, the number of possible states for each component in the reconstructed sequence is $m! =6$.

### Transfer entropy

TE (Transfer entropy) is a non-parametric method based on information theory that can be used to efficiently compute the directional information flow between two EEG sequences^27,28^. Therefore, it is often used to construct connectivity networks of EEG, allowing us to obtain causal relationships of different brain regions. The TE from one time series Y to another one X is defined as:

$$T_{Y \rightarrow X} = \sum p(x_{n+1}, x_n^{(k)}, y_n^{(l)}) \log \left( \frac{p(x_{n+1} \mid x_n^{(k)}, y_n^{(l)})}{p(x_{n+1} \mid x_n^{(k)})} \right)$$

Obviously, the TE from *X* to *Y* is not necessarily equal to its from *Y* to *X* (it is usually unequal). Therefore, by calculating the TE between different channels of interest, we can obtain the adjacency matrix that represents the flow of unequal information between these regions, which in turn allows further analysis of the corresponding network characteristics.

### Node degree

Node degree is a key characteristic in graph theory that quantifies the importance of a node within a network. It is usually defined as the number of edges connected to a node. Since the connectivity network constructed based on transfer entropy in this study is a directed weighted network, the out and in degree are defined as:

$$d_{\text {out}}(u) = \sum _{(u,v) \in E} w(u,v)$$

$$d_{\text {in}}(u) = \sum _{(u,v) \in E} w(v,u)$$

where $d_{out}$ is the out degree of a node, denoting the sum of the weights of the information flows originating from this node. $d_{in}$ is the in degree of a node, denoting the sum of the weights of the information flows arriving at this node^29^. A larger node degree usually indicates greater involvement in the network.

## Results

### Time-frequency analysis

**Fig. 2:**
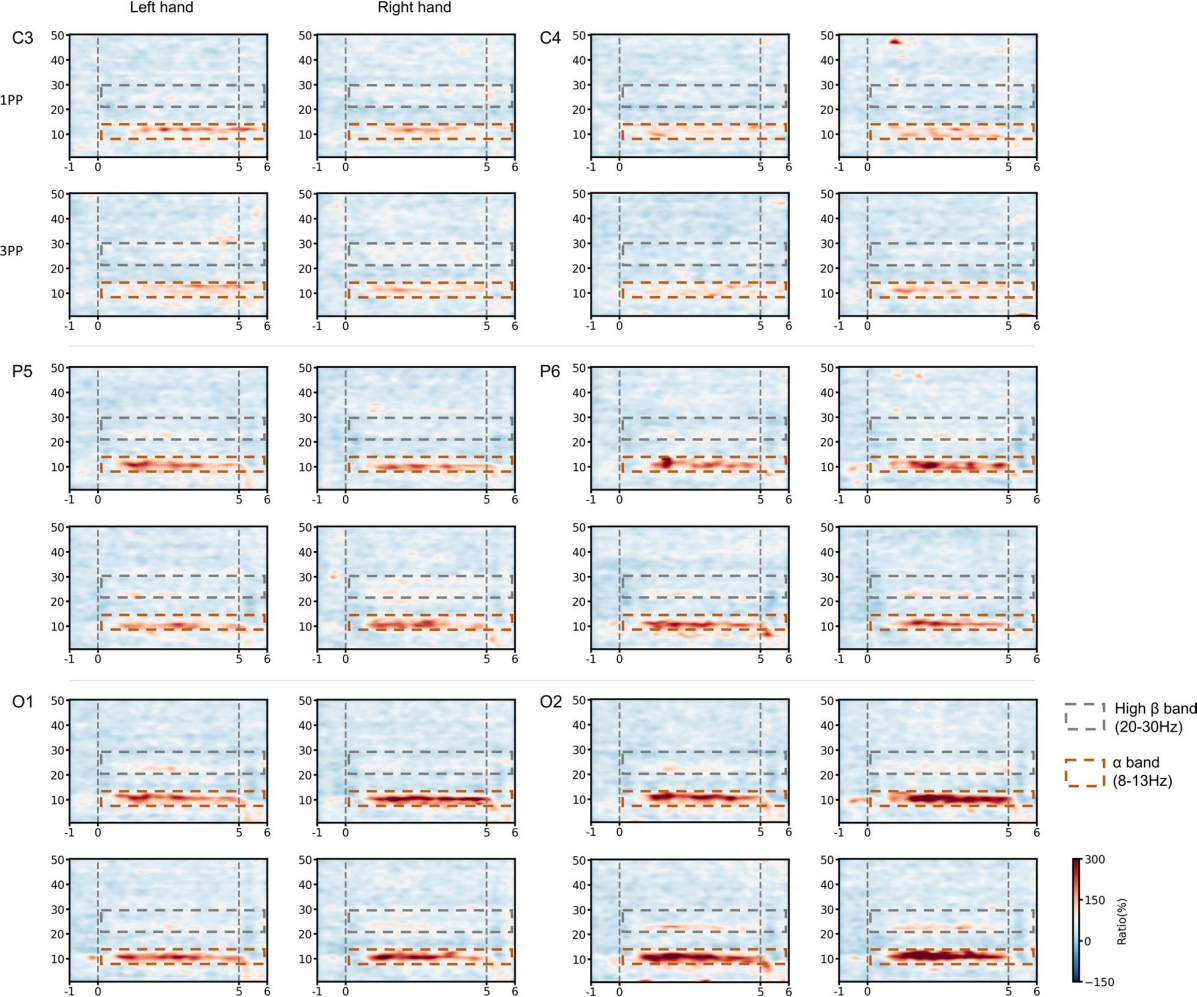
Event related power spectrum of 6 channels under 4 task conditions, using wavelet transform over the frequency range of 0.1-50 Hz, time window of -1-6s.

Fig. 1 illustrates the ERD/ERS of six channels. Across all experimental conditions and channels, a significant alpha-band (8–13 Hz) ERS occurred approximately 1 second after the onset of imagery and persisted until the end of tasks. Additionally, a relatively weak high beta-band (20–30 Hz) ERS was observed (Fig. 2).

It is worth noting that none of the above between-group comparisons of the grand-averaged ERS showed statistically significant differences (Wilcoxon signed-rank test, p<0.05).

### Connectivity network

#### Multi-scale permutation symbolization

Multi-scale analysis was performed on the two frequency bands identified in the time-frequency analysis. The selection of the scale factor was based on two considerations. First, it needed to ensure that the length of the coarse-grained sequences met the requirements for subsequent PSR and TE calculations. Second, it had to be appropriate in terms of the physical meaning of PSR. In this study, the delay factor of PSR was determined using the autocorrelation function method. As the scale factor increased, the delay factor decreased until it reached 1. If the scale factor was too large, even with a delay factor of 1 (resulting in a highly compact reconstruction), there was a risk of losing essential dynamic information of the original system.

To reflect TE differences at different scales, we selected scale factors of 4 and 16 for the alpha band, capturing both small-scale factors that retain more details and large-scale factors that filter out abundant details. For the high beta band, we selected scale factors of 3 and 8 following the same principle. Next, we applied PSR-based permutation symbolization to the coarse-grained sequences in both frequency bands, obtaining the state sequences required for TE calculations.

#### Transfer entropy

**Fig. 3:**
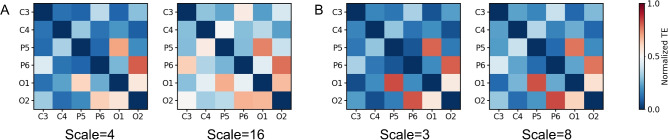
The Adjacent matrices in (**A**) alpha band (scale = 4 and 16) and (**B**) high beta band (scale = 3 and 8).

For the six channels of interest, adjacency matrices were computed at different scale factors (Fig. 3). In these matrices, elements represent connections between different channels, and their colors correspond to the normalized TE values, averaged across all subjects.

The connectivity networks suggested common patterns across all four scales and both frequency bands. First, they consistently showed a dominant information flow between two pairs of same-hemisphere brain regions, P5–O1 and P6–O2. Second, significant information flow was also observed between O channels across hemispheres as well as between P/O channels and their contralateral C channels. At scale 4 in the alpha band and scales 3 and 8 in the high beta band, the information flow in P5–O1 and P6–O2 was particularly dominant. However, at a larger scale (scale 16, alpha), the proportion of other information flows increased.

**Fig. 4:**
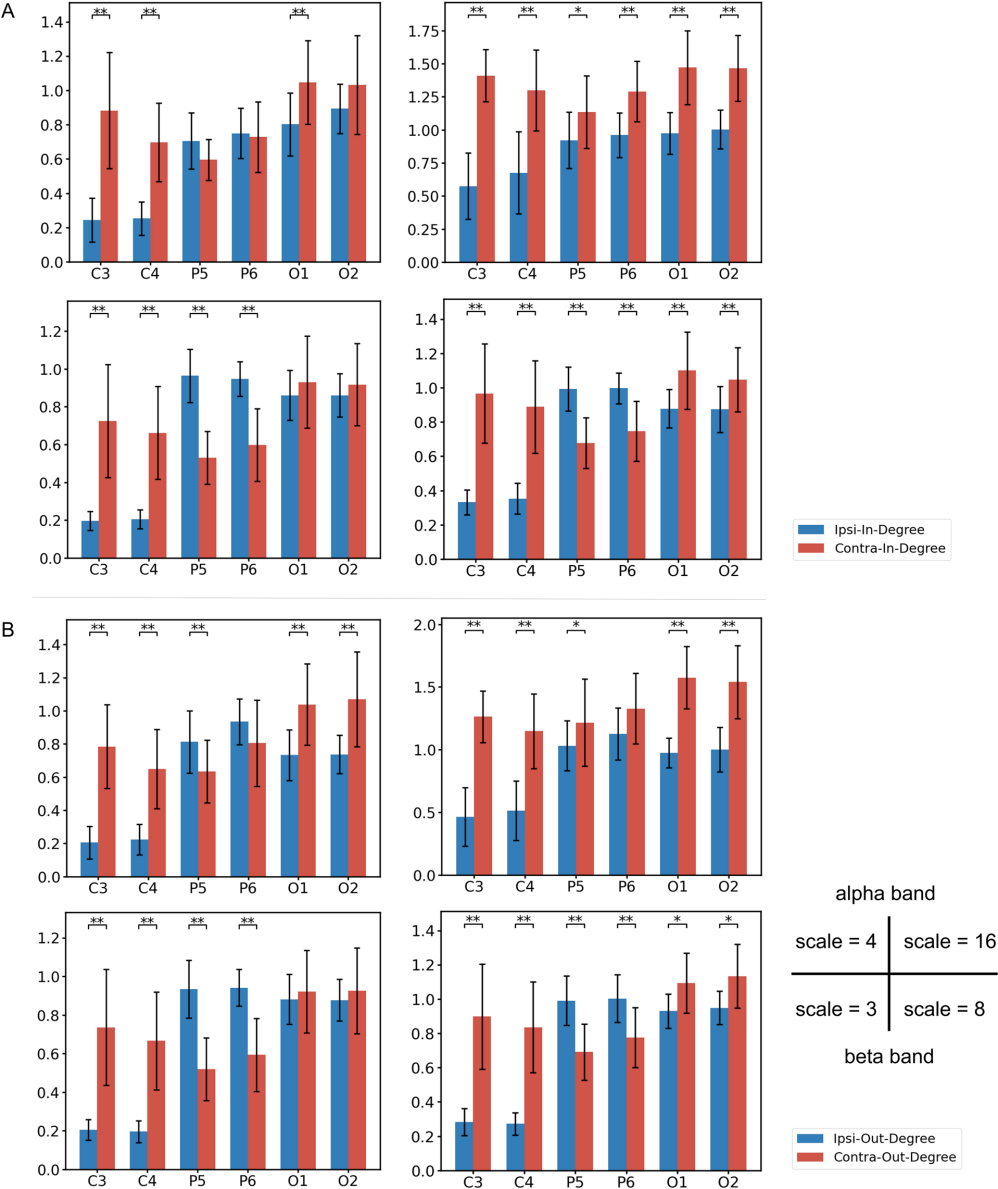
(**A**) In and (**B**) out degree of each node, distinguished by the information flow of ipsilateral and contralateral. * and ** suggest significant level under 0.05 and 0.01, respectively (N=17, Wilcoxon signed-rank test).

Network node degree analysis further revealed information flow patterns centered on each channel (Fig. 4). For C3 and C4, significant contralateral-dominated inflows and outflows were observed in all conditions. For P5 and P6, contralateral information flow exceeded ipsilateral flow in the alpha band at scale 16, whereas other conditions showed an ipsilateral-dominated transfer pattern. For O1 and O2, contralateral information transfer dominated across all conditions.

### Hand effect

A comparison of TE networks across experimental conditions revealed significant information flow differences based on hand and perspective effects (N=34, Wilcoxon signed-rank test) (Table 1 and Table 2).

**Table 1:** Significant changes in information flow between hand groups. * and ** suggest significant level under 0.05 and 0.01, respectively.

**Frequency band**	**Scale**	**Flow**	**Left**	**Right**	**p-value**
alpha	4	P5 $\rightarrow$ C4	0.3525	0.2984	0.0007**
		P6 $\rightarrow$ O1	0.2502	0.2610	0.0330*
	16	C3 $\rightarrow$ P6	0.5171	0.5726	0.0391*
		C4 $\rightarrow$ P5	0.5258	0.4248	0.0146*
high beta	3	O2 $\rightarrow$ C4	0.0604	0.0466	0.0238*
	8	C4 $\rightarrow$ P6	0.1771	0.1401	0.0464*
		O1 $\rightarrow$ C3	0.0941	0.1260	0.0374*

**Table 2:** Significant changes in information flow between perspective groups. * and ** suggest significant level under 0.05 and 0.01, respectively.

**Frequency band**	**Scale**	**Flow**	**1PP**	**3PP**	**p-value**
alpha	16	C3 $\rightarrow$ P6	0.6010	0.5101	0.0187*
		C3 $\rightarrow$ O1	0.2201	0.1735	0.0286*
		C4 $\rightarrow$ O1	0.3904	0.2830	0.0299*
		C4 $\rightarrow$ O2	0.1984	0.1561	0.0227*
		P5 $\rightarrow$ O1	0.7960	0.7262	0.0169*
		P6 $\rightarrow$ C3	0.6727	0.5912	0.0187*
		P6 $\rightarrow$ O2	0.7979	0.7750	0.0391*
high beta	3	C3 $\rightarrow$ P5	0.1404	0.1386	0.0328*
		P6 $\rightarrow$ O1	0.1487	0.1833	0.0484*
		P6 $\rightarrow$ O2	0.7856	0.8392	0.0015**
		O2 $\rightarrow$ P6	0.8263	0.8559	0.0028**
	8	P6 $\rightarrow$ O1	0.2115	0.2583	0.0261*
		P6 $\rightarrow$ O2	0.7541	0.7850	0.0216*

**Fig. 5:**
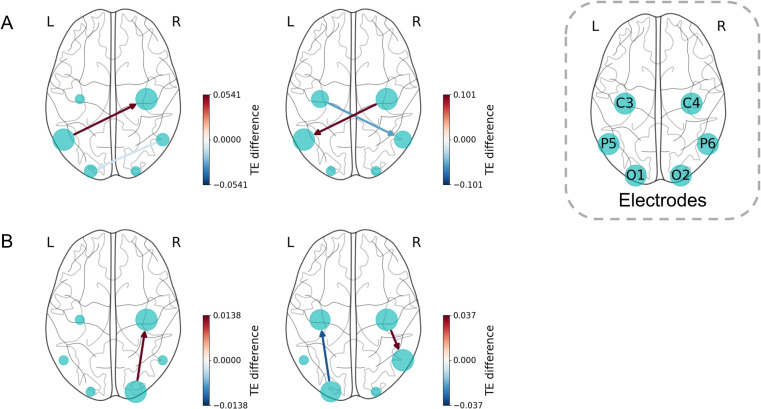
Directed connectivity with significant hand main effect for four scales of (**A**) alpha band and (**B**) high beta band. The edges’ color corresponds to their weights, which are the TE differences between the groups (averaged value of left-hand group – right-hand group). Red edges denote left-hand group > right-hand group (positive value). Blue edges denote right-hand group > left-hand group (negative value).

In the alpha band at both scales, there are information flows that the left-hand group was significantly greater than the right-hand group between P5-C4. Notably, at a small scale, the dominant information flow direction was P5 $\rightarrow$ C4, whereas at a large scale, it was C4 $\rightarrow$ P5. Additionally, at scale 4, P6 $\rightarrow$ O1 showed weaker information flow in the left-hand group than in right-hand group. At scale of 16, similar trend was observed for C3 $\rightarrow$ P6 (Fig. 5).

In the high beta band, information flow between C4 and its ipsilateral parietal-occipital region was greater in the left-hand group. Additionally, significant information flows were observed from O2 $\rightarrow$ C4 at scale 3 and from C4 $\rightarrow$ P6 at scale 8. Moreover, information flow of O1 $\rightarrow$ C3 showed significant at scale 8.

### Perspective effect

**Fig. 6:**
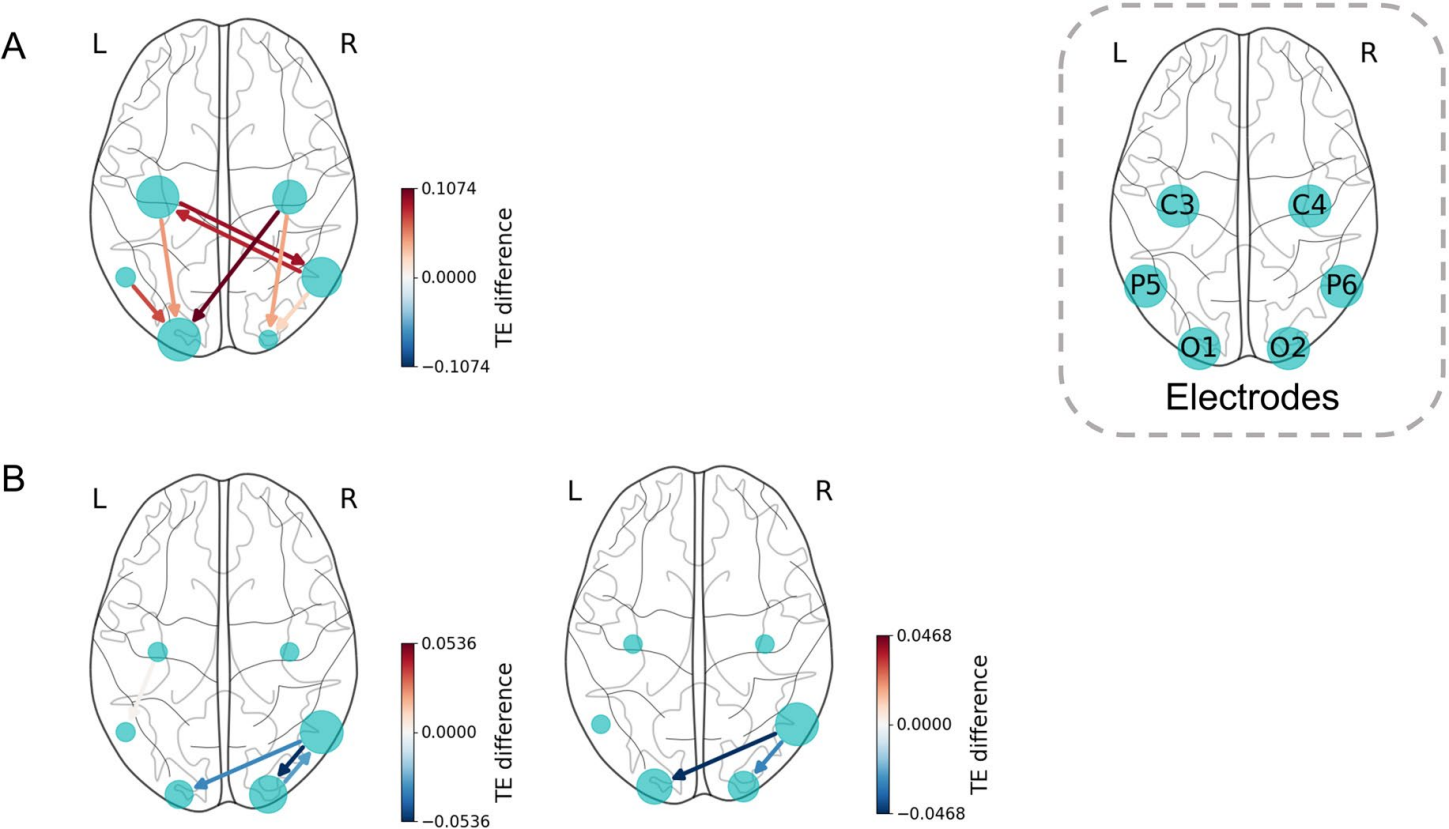
Directed connectivity with significant perspective main effect for three scales of (**A**) alpha band and (**B**) high beta band.

In the alpha band at scale 4, no significant differences in information flow were observed between groups. In contrast, at scale 16, anterior-to-posterior information flow was significantly greater in the 1pp group than in the 3pp group. This included information flows from bilateral C and P nodes to ipsilateral O nodes, C4 $\rightarrow$ O1, and bidirectional flows between C3 and P6 (Fig. 6).

In the high beta band, the 3pp group exhibited significantly greater information flow than the 1pp group in the right parietal-occipital region. At scale 3, bidirectional flows were observed between P6 $\rightarrow$ O1 and P6 $\rightarrow$ O2, as well as from C3 $\rightarrow$ P5. At scale 8, the significant information flows were P6 $\rightarrow$ O1 and P6 $\rightarrow$ O2.

## Discussion

Research on brain activity during VMI has been comparatively limited as the KMI paradigms are used as templates. However, numerous studies have indicated the critical role of the posterior parietal and occipital regions in encoding motor intentions and participating in visual imagery^30–32^, which suggests the potential for applying VMI in further applications. In this study, we first confirmed the power spectrum characteristics of VMI using time-frequency analysis, a widely adopted method for capturing the macroscopic patterns of MI EEG. Building on this, we investigated directional connectivity within relevant frequency bands. For the first time, a multi-scale transfer entropy-based approach was employed to examine the effective connectivity network of motor imagery EEG.

Unlike the well known time-frequency characteristics of KMI-EEG, there remains no consensus regarding VMI-EEG. This bias may arise from variations in experimental paradigms and spontaneous EEG activity among subjects, making it difficult to control all influencing factors. Moreover, the conventional model in which KMI suppresses neural synchronization in the motor cortex may not be directly applicable to VMI. While some studies have reported ERD phenomena in the parietal or occipital lobes during VMI^10,33^, our study did not observe such effects. This discrepancy may be attributed to differences in the sensory modalities emphasized by the experimental design^34^. Specifically, the extent to which subjects perceive imagery as either ’motor sensations’ or ’visual experiences’ may influence neural activity. The former likely engages pre-existing motor templates (linked to motor execution), while the latter aligns more closely with visual perception (related to motor observation) and contains fewer motor-perceptual components^3,6^. Given that our study utilized video as a guiding stimulus, EEG signals from the parietal-occipital regions during imagery exhibited consistent ERS. This suggests that emphasizing explicit visual sensory attributes in the task effectively isolates kinesthetic experiences from motor imagery.

Our network analysis supports previous findings that the posterior parietal and occipital regions, rather than the motor cortex, play a dominant role in VMI^2,3,35,36^. At a macroscopic level, the VMI process was primarily characterized by strong information flow between the ipsilateral inferior parietal and occipital lobes, as well as bilateral occipital regions. Additionally, bidirectional connections between the parietal and motor regions (primarily contralateral) were observed at various scales.

The integration of multi-scale analysis with PSR revealed novel insights into directional connectivity, including previously unreported phenomena. In the analysis of the hand effect, we found consistent intergroup differences in both the alpha and high beta bands. However, directional connections differed across scales, with C4 $\rightarrow$ P5 in the alpha band and C4 $\rightarrow$ P6/O2 in the high beta band. This suggests the different time dependencies within information exchanges between these regions, which are difficult to capture using conventional symbolization methods applied to homogeneous time series.

Furthermore, directional connectivity involving motor area channels C3 and C4 followed a distinct contralateral pattern and exhibited a significant hand effect. However, no such pattern was observed for the perspective effect. Notably, in the alpha band, this effect was confined to interactions between motor and posterior parietal regions and did not extend to the occipital regions. In contrast, in the high beta band, it included direct connectivity between motor and occipital regions. These findings indicate that the hand effect modulates visual/spatial attention in posterior parietal and occipital regions through motor-related processes but does not influence the basal excitation of the visual cortex^10,37^.

As for the analysis of the perspective effect, in the alpha band, imagery from the 1pp perspective showed significantly greater backward information flow, particularly toward the occipital region, compared to 3pp imagery. Although the VMIQ-2 results did not indicate differences in the subjective vividness of the two perspectives (1pp: 1.86 ± 0.74, 3pp: 1.81 ± 0.63), directional connectivity analysis revealed enhanced top-down modulation centered in the occipital lobe for 1pp imagery^38–40^. This process involves direct and indirect (posterior parietal-mediated) task-related modulation from the motor region, along with spatial sensory regulation from the posterior parietal region, collectively enhancing visual experience generation in the occipital cortex^30,41,42^.

In the high beta band, 3pp imagery showed stronger information flow from the right posterior parietal lobe to the occipital lobe compared to 1pp imagery. This suggests the unique role of the right posterior parietal region in modulating occipital activity from an external perspective through attention-based spatial localization^43–46^.

This study provides new insights into the effective connectivity of VMI EEG, demonstrating the dominant role of posterior parietal and occipital regions play in motor imagery processing. By applying a multi-scale transfer entropy approach, we revealed unique connectivity patterns that depend on both the hand effect and perspective effect, suggesting underlying mechanisms for different types of motor imagery strategies. These findings contribute to a deeper understanding of the neural dynamics of VMI, but several limitations should be clarified.

First, although the primary objective of this study was to investigate directed interactions among specific ROIs, we should also emphasize the importance of discovering large-scale network dynamics, as increasingly reported in recent whole-brain connectivity studies^47,48^. Our focus was on capturing fine-grained modulations within regions most closely associated with VMI and constructing as well as analyzing a full 64 $\times$ 64 directed network would not only be computationally demanding but also less practical for application-oriented EEG interfaces. Therefore, we adopted a hypothesis-driven region-of-interest (ROI) approach, targeting visual, parietal, and motor areas previously implicated in the VMI literature. Future studies combining high-density EEG with simultaneous fMRI may extend these findings to a truly whole-brain scale regional coordination patterns.

Second, due to the potential bias introduced by non-high-density EEG in source-level analysis^49^, this study employed a scalp-level approach for computation and analysis. Although this method retains some risk of source leakage due to volume conduction of EEG, the combination of permutation symbolization and non-zero embedding delays ($\tau$ = 8-16 ms) effectively suppresses connections arising from zero-lag coupling^27,50^. Additionally, the use of a common average reference helps mitigate reference-related bias.

Finally, as discussed earlier, this study employed an experimental paradigm that explicitly constrained the subjects’ imagery templates. While this approach helped control experimental variables, it also overlooked the variability in individual imagery strategies. In real-world motor imagery applications, factors such as the clarity of goal direction, the sensory attributes emphasized during imagery, and the subject’s prior experience with the action can all influence imagery strategies, leading to distinct brain activity patterns. Therefore, further exploration of task-independent and generalized brain connectivity during VMI is essential for enhancing the applicability of VMI-EEG in practical scenarios.

## Data Availability

The data presented in this study are available from the corresponding author upon request. The data are not publicly available due to restrictions for participant privacy.
